# Investigation of Electrochromic, Combinatorial TiO_2_-SnO_2_ Mixed Layers by Spectroscopic Ellipsometry Using Different Optical Models

**DOI:** 10.3390/ma16124204

**Published:** 2023-06-06

**Authors:** Noor Taha Ismaeel, Zoltán Lábadi, Peter Petrik, Miklós Fried

**Affiliations:** 1 Institute of Technical Physics & Materials Science, Centre for Energy Research, Konkoly-Thege Rd. 29-33, 1121 Budapest, Hungary; noor.t@ilps.uobaghdad.edu.iq (N.T.I.); labadi.zoltan@ek-cer.hu (Z.L.); petrik.peter@ek-cer.hu (P.P.); 2 Doctoral School on Materials Sciences and Technologies, Óbuda University, 1034 Budapest, Hungary; 3 Institute of Laser for Postgraduate Studies, University of Baghdad, Baghdad 10070, Iraq; 4 Department of Electrical Engineering, Institute of Physics, Faculty of Science and Technology, University of Debrecen, 4032 Debrecen, Hungary; 5 Institute of Microelectronics and Technology, Óbuda University, Tavaszmezo Str. 17, 1084 Budapest, Hungary

**Keywords:** Titanium-Tin oxide, reactive sputtering, spectroscopic ellipsometry, electrochromic materials

## Abstract

We determined the optimal composition of reactive magnetron-sputtered mixed layers of Titanium oxide and Tin oxide (TiO_2_-SnO_2_) for electrochromic purposes. We determined and mapped the composition and optical parameters using Spectroscopic Ellipsometry (SE). Ti and Sn targets were put separately from each other, and the Si-wafers on a glass substrate (30 cm × 30 cm) were moved under the two separated targets (Ti and Sn) in a reactive Argon-Oxygen (Ar-O_2_) gas mixture. Different optical models, such as the Bruggeman Effective Medium Approximation (BEMA) or the 2-Tauc–Lorentz multiple oscillator model (2T–L), were used to obtain the thickness and composition maps of the sample. Scanning Electron Microscopy (SEM) with Energy-Dispersive X-ray Spectroscopy (EDS) has been used to check the SE results. The performance of diverse optical models has been compared. We show that in the case of molecular-level mixed layers, 2T–L is better than EMA. The electrochromic effectiveness (the change of light absorption for the same electric charge) of mixed metal oxides (TiO_2_-SnO_2_) that are deposited by reactive sputtering has been mapped too.

## 1. Introduction

Metal oxides are widely studied with respect to their electrochromic behavior and properties for applications such as display devices and smart windows. To decrease the absorbed heat in buildings, electrochromic films have been used as smart windows for the preservation of glass windows from extra heating [1]. Electrochromic materials have been applied in energy-effective vitrification, automobile sunroofs, smart windows, and mirrors. Transition-metal oxides such as titanium, tungsten, nickel, vanadium, and molybdenum oxides have been considered the most promising electrochromic materials [2]. The formation of a smart window contains an electrochromic material layer (usually metal oxide layers) sandwiched between transparent conductive layers and some solid electrolytes. 

To turn transparent glass opaque and back to the transparent state, a low electric current is used. The transmittance can be controlled by modifying the optical properties [3]. The protection from heat radiation through the glass would be obtained by using coatings on glass made by films from semiconductor metal oxide such as Tungsten trioxide (WO_3_), TiO_2_, Chromium (II) oxide (CrO), Nickel (II) oxide (NiO), Niobium (V) oxide (Nb_2_O_5_), Iridium (IV) oxide (IrO_2_) [3], and Molybdenum trioxide (MoO_3_) [4,5]. 

Typically, nanoscale oxides are considered according to their high thermal conductivity, low thermal expansion coefficient, and insulation. The application of this type of coating gives an advanced surface quality. The heat transfer rate and thermal conductivity are increased due to the increases in the concentration of nanoparticles [6,7].

Several methods of deposition can be considered: sputtering [8], sol-gel method [4], sintering [9], Atmospheric Pressure Chemical Vapor Deposition (APCVD) [10], and dipping [11]. 

Titanium oxide films were sputter-deposited in a non-aqueous medium, spray deposited from reactive sputtering. Chronoamperometric experiments associated with transmittance spectra in LiClO_4_-propylene carbonate solutions were carried out and compared to the optical properties of titanium oxide films with different stoichiometries [12]. SE is a high-accuracy optical characterization technique [13]. 

Many researchers have used SE for pure or combinatorial material investigation [14,15,16,17,18,19,20,21]. The combinatorial approach used to investigate mixed metal oxides has several advantages. Fried et al. [22] have used SE (which is a fast, cost-effective, and non-destructive method) for the investigation and mapping of WO_3_-MoO_3_ mixed layers after sputtering. Different optical models, such as EMA and 2T–L, have been used to achieve the composition map and thickness map of the sample layers. While pure TiO_2_ was investigated as an electrochromic material [23], SnO_2_ or TiO_2_-SnO_2_ [24,25] mixtures were studied only as photocatalytic materials. There is no such publication where pure SnO_2_ or TiO_2_-SnO_2_ mixtures are studied as electrochromic material.

During this work, reactive magnetron sputtering (in Ar-O_2_ plasma) has been used to produce all combinations (from 0 to 100%) of TiO_2_-SnO_2_ mixed layers on silicon wafers. The sample preparation time took 4 h in the vacuum chamber, including the vacuum-preparation time. By using the combinatorial process, all the compositions (from 0 to 100%) have been achieved in the same sputtering chamber after one sputtering. SEM with EDS has been used to check the SE results.

The objective of this work was to investigate the electrochromic effectiveness (the change of light absorption for the same electric charge) of TiO_2_-SnO_2_ mixed layers in a wide compositional range. We expected that using metal atoms with different diameters in the layers would have a positive effect.

## 2. Materials and Methods

**Figure 1:**
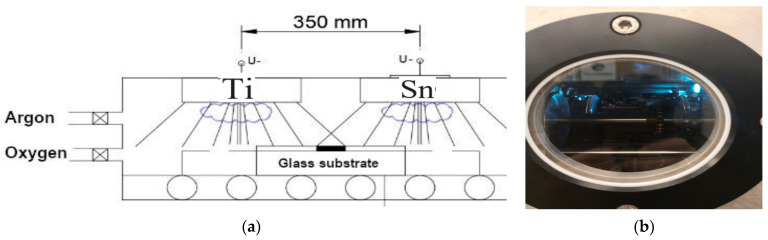
(**a**) arrangements of the two targets in a closer position (35 cm from each other); (**b**) the chamber for the DC magnetron sputtering device after being air vacuumed. Blue light is from the Ar-O_2_ plasma.

In the chamber of magnetron sputtering, as demonstrated in Figure 1b, the layers were deposited in a reactive (Ar + O_2_) gas mixture in a ~2 × 10^−6^ mbar high vacuum, where the pressure of the process was ~10^−3^ mbar. Around 30 sccm/s Ar and 30 sccm/s O_2_ volumetric flow rate was applied inside the chamber. The substrates were 4-inch diameter IC-grade and 3-inch diameter, highly conductive (0.001 Ωcm) Si-wafers.

The movement speed was 5 cm/s (back and forth) in the geometry, which can be seen in Figure 1. By using this combinatorial process, all the compositions (from 0 to 100%) were achieved in the same sputtering chamber after one sputtering. Around 50–50% composition can be expected in the middle of the specimen. The Si-wafers and control Si-stripes were placed on a 30 cm × 30 cm glass, as shown in Figure 1a. The changing composition area is around the center between the two targets, and the Si samples were placed there. The power of the plasma was in the range of 0.75–1.5 kW for the two targets and was independently controlled. About 300 walking cycles were applied with 5 cm/s movement speed. 

Figure 1a shows that the sputtering targets were placed 35 cm from each other. According to the measurements, the two sputtered material fluxes ‘material streams’ overlapped around the center. The Metal/Oxygen atomic ratio in the layers was 1:2 at the applied oxygen partial pressure according to the SEM–EDS analysis technique.

**Figure 2:**
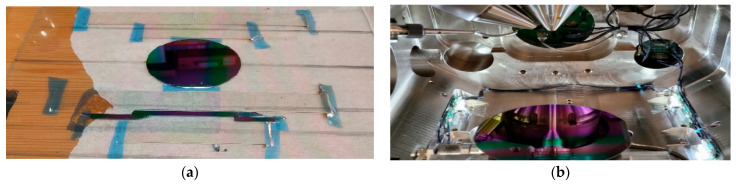
(**a**) Graded TiO_2_–SnO_2_ layer on 3-inch Si (circular sample, upper) and the Si-stripe samples, lower; (**b**) Combinatorial TiO_2_-SnO_2_ layer on a 4-inch Si-wafer in the SEM-chamber (Dual-beam SEM + FIB Thermo Scientific Scios2).

The optical mapping [21] was performed using Woollam M2000 SE, and the measurements were evaluated with the CompleteEASE v. 5.15 software [26]. To obtain the mapping parameters, oscillator functions and compact optical models were used. The applicability of the optical model can be judged from the value of the Mean Squared Error (MSE), so a lower MSE indicates a better fit because of the difference between the curves. [13] The silicon wafers and Si-stripes (Figure 2a) were used for SEM and Dual-beam SEM + FIB Thermo Scientific Scios2, with EDS measurements as well (Figure 2b). The Ti/Sn ratio was calculated point-by-point to compare and validate the results of the SE evaluation.

The coloration process was followed in real-time at the central point of the 3-inch diameter highly conductive (0.001 Ωcm) Si-wafer. Electrochemical measurements were performed in a liquid cell filled with a 1 M lithium perchlorate (LiClO_4_)/propylene carbonate electrolyte, and a Pt wire counter electrode was placed into the electrolyte alongside a reference electrode. A controlled current was applied through the cell during a 4 min coloration.

**Figure 3:**
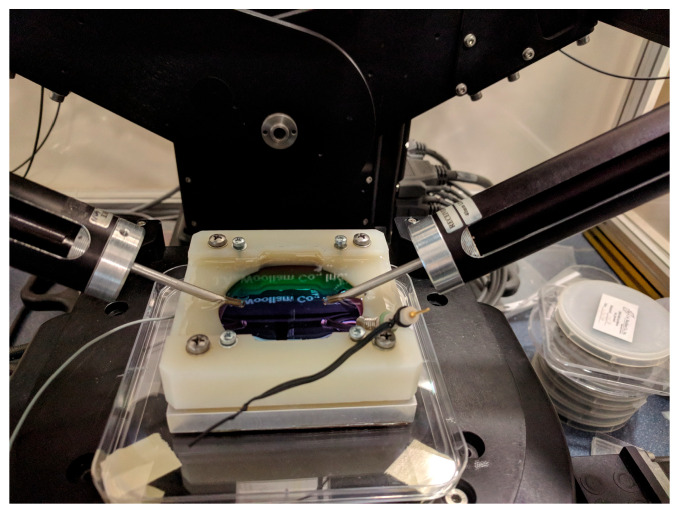
Combinatorial TiO_2_–SnO_2_ layer on highly conductive 3-inch Si-wafer in an electrochemical fluid cell during in situ, real-time SE measurements.

After the coloration process, the whole sample (in the dry state) was mapped by SE. The edges were under the Teflon cover (during the coloration process) so that only the central 6 cm diameter part was measured, as demonstrated in Figure 3. 

## 3. Results

The physical combination of the TiO_2_ and SnO_2_ in the mixed layers can be considered as a mixture of distinct phases or as an atomic-scale mixture. Our aim was to determine (point-by-point) the volume fraction of each constituent. If we consider it as a mixture of distinct phases, then we should use the Bruggeman Effective Medium Approximation (BEMA) [27]. Equation (1) shows BEMA, where the constituents are considered coequal.

0 = ∑f_i_(ε_i_ − ε)/(ε_i_ + 2ε), (1)

where ε is the effective complex dielectric function of the composite layer; f_i_ and ε_i_ denote the volume fraction and the complex dielectric function of the ith component. In the case of two components, the equation formula is a complex quadratic, where the unknown is the effective dielectric function (ε), and we can select the good solution, as the wrong solution is physically meaningless. Dielectric functions of the two constituents were determined from the extreme edges of the Si-stripes where the TiO_2_ and SnO_2_ are in a pure format.

If we consider the mixture as an atomic-scale mixture, the Tauc–Lorentz (T–L) oscillator model is more appropriate. The T–L model is a combination of the Tauc and Lorentz models [28]. The Lorentz model is a classical model where an electron is bound to an ionic core with a spring. If the light is shone, it will induce dielectric polarization. The Lorentz model assumes that the electron oscillates in a viscous fluid. As the mass of the electron is far lesser, the position of the ionic core is fixed. So, the Lorentz model (and its modified version, the Tauc–Lorentz oscillator model) considers each “molecule” as an individual damped oscillator. The dielectric function of the mixed material can be considered as a summation of the elementary oscillators of the TiO_2_ and SnO_2_ “molecules”. The Amplitude-ratio of the two elementary oscillators can be considered as the atomic ratio of the Ti and Sn atoms.

The Tauc–Lorentz (T–L) oscillator model contains four parameters: transition amplitude (oscillator strength), broadening coefficient of the Lorentz oscillator, peak position for the Lorentz oscillator, and band gap energy (E_g_), which is taken to be the photon energy where ε_2_(E) reaches zero. When the E photon energy is less than the bandgap energy, E_g_, ε_2_(E) is zero. The real part of the dielectric function ε_1_(E) can be obtained from ε_2_(E) through the Kramers–Kronig relation.

In the mixed layers, five fitting parameters were used: two amplitudes for each material (oscillator strengths), interface and surface roughness thicknesses, and the main layer thickness. The optical model for the dry samples consisted of 3 layers: interface layer, TiO_2_ + SnO_2_ mixed layer, and surface roughness layer. The interface layer (between the Si-substrate and the mixed layer) proved less than 15 nm, while the surface roughness layer proved less than 5 nm. We used the measurements near the edges of the samples (pure component materials) to determine the fundamental parameters (band gap energies, the broadenings and the peak positions) for the two materials. 

For the electrochromic measurements, where the light absorption was measured in the visible wavelength region over 400 nm, we used the simple Cauchy formula to describe the complex refractive as in Equations (2)–(4):

N = n + ik,(2)

where i is the imaginary unit, k is the imaginary part (extinction), N is the complex refractive index, and n is the real part of N.

n(λ) = A + B/λ^2^ + C/λ^4^, (3)

k(λ) = ke ^U(1239.84/λ − Eb),^ (4)

where U, A, B, C, and k are the fitted parameters. The complex dielectric function (ε) and the complex refractive index (N) are coequal, as in the Equations (5)–(7):

(ε) = ε_1_ + iε_2_ = N^2^, (5)

ε_1_ = n^2^ − k^2^, (6)

ε_2_ = 2nk. (7)

To evaluate the real-time measurement, we used a 2-layer optical model with the Cauchy dispersion. To estimate the change after the colorization process, we used a simple 1-layer optical model with the Cauchy dispersion.

### Comparison of the Optical Models

**Figure 4:**
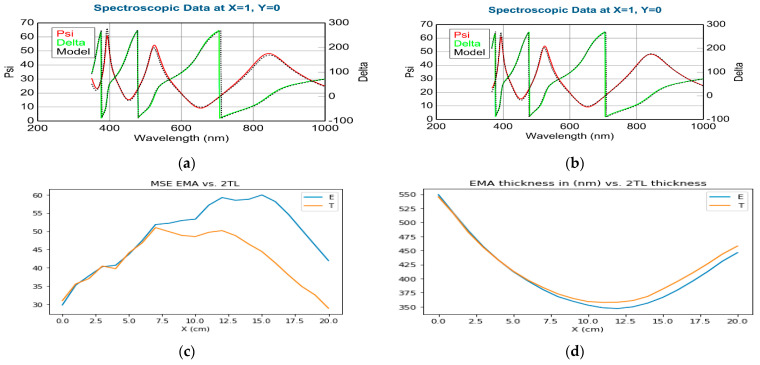
Comparison of (**a**) EMA; (**b**) 2T–L modelling (TiO_2_-SnO_2_); (**c**) MSE for EMA (the blue curve) vs. 2T–L (the orange curve), (**d**) is the thickness (EMA (the blue curve) vs. 2T–L (the orange curve) by home-made software version 1.0 coded in Python version 3.11 language.

**Figure 5:**
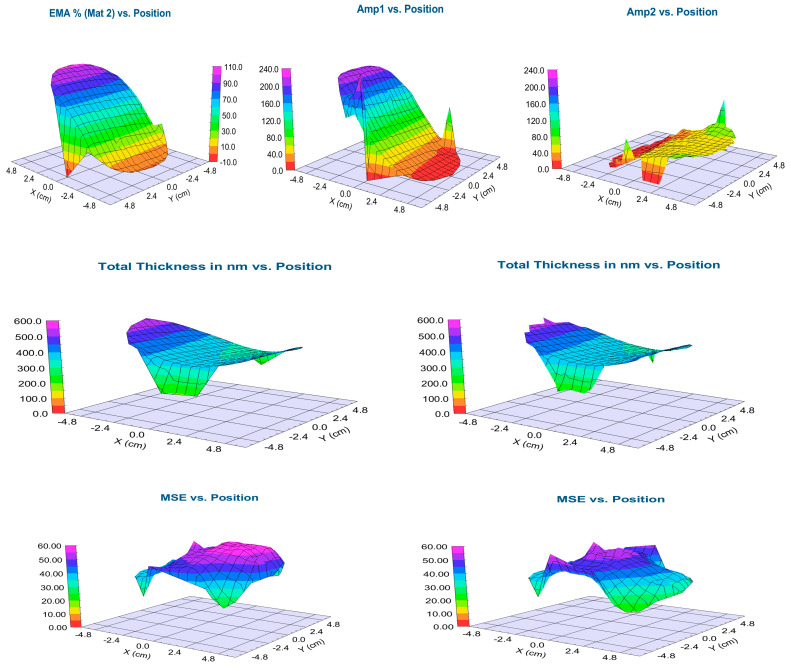
Shows TiO_2_–SnO_2_ maps from the 4 inch-wafer by BEMA modelling (**left**) 2T–L modelling (**right**), upper row: EMA% (**left**) and Tauc–Lorentz Amplitudes (**right**), middle row: total thickness maps, lower row: MSE maps (showing that the 2T–L model is better, the MSE values are lower). Pictures were made by the CompleteEASE v. 5.15 software.

We applied the 2T–L and the BEMA optical model to evaluate the mapping measurements on the Si-stripes (shown in Table 1 and Figure 4) and the 4-inch Si-wafer (shown in Figure 5). Both modeling processes gave good results, as shown in Figure 4 where the measured Psi and Delta spectra are in good agreement with the Model calculations. However, one can see that the MSE (Mean Squared Error) is significantly lower for the 2T–L model, especially around the 50–50% composition, as demonstrated in Figure 4c and Figure 5 in the lower row. The calculated thickness values are not significantly different, as evident in Table 1, Figure 4d and Figure 5 in the middle row. The difference (less than 2%) in the thickness values can be explained by the different optical models and does not change the conclusion.

**Figure 6:**
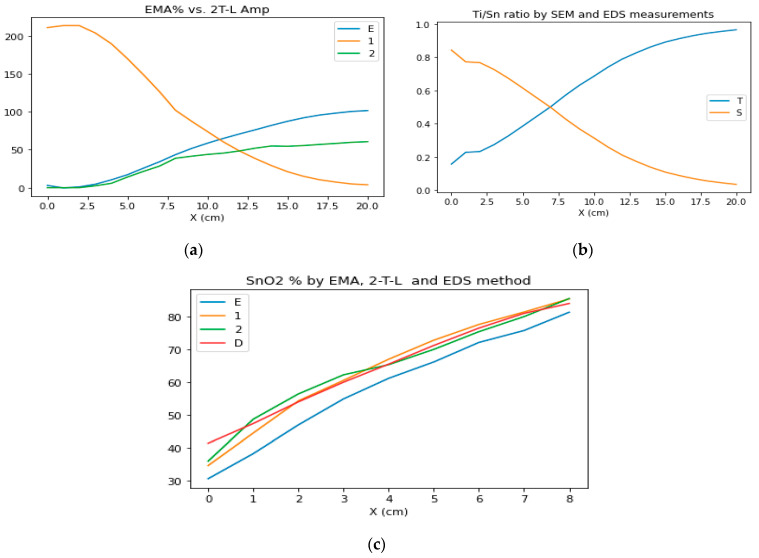
TiO_2_–SnO_2_ ratio curves from SE and SEM–EDS measurements at the center line of the 4-inch sample; (**a**) the blue curve for EMA% (E), the orange curve for Amp1 (TiO_2_), the green curve for Amp2 (SnO_2_); (**b**) Ti/Sn ratio from SEM–EDS measurements, (the blue curve for Ti ratio and the orange curve for Sn ratio); (**c**) SnO_2_% derived from EMA% (the blue curve, E), 2T–L models (the orange curve for Amp1 and the green curve for Amp2), the red curve for EDS% measurements (by home-made software coded in Python language).

Figure 5 shows the mapping results of the 4-inch wafer, which shows similar results to the results on the Si-stripes. The values change only in the X-direction (where the samples were moved during the sputtering process, as shown in Figure 1), while the values do not change in the Y-direction. The EMA % (Figure 5 upper row left) shows the calculated volume fraction of the TiO_2_ from the BEMA model, while Amp1 and Amp2 show the oscillator strengths of the TiO_2_ and SnO_2_ from the 2T–L calculations. One can see that the normalized amplitude values can be used as a good approximation for the composition at a given point of the sample. We compared the EMA % values and the composition values calculated from the normalized amplitude values with the results of the SEM–EDS in Figure 6c. The calculated thickness values are not significantly different, as demonstrated in the middle pictures in Figure 5.

## 4. Discussion

We validated the results of the SE modeling with SEM–EDS measurements, as demonstrated in Figure 6b. Figure 6a shows the EMA% (MAT2-SnO_2_%, blue line E) values from the BEMA model and the Amp1 (TiO_2_ oscillator strength) and Amp2 (SnO_2_ oscillator strength) from the 2-Tauc–Lorentz (2T–L) model. Figure 6c shows the results together, where we normalized the Amp1 and Amp2 to 100%. One can see the good agreement between the SEM–EDS and the 2T–L results.

### Electrochromic Measurements

**Figure 7:**
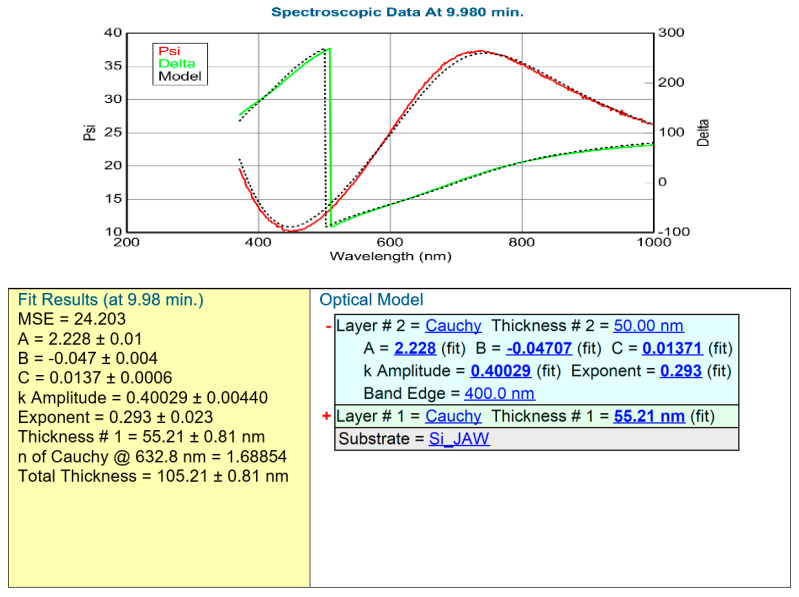
Typical example of a fitted SE spectrum for the details of the model structure; SE spectra were evaluated using a multi-layer, multi-parameter optical model applying two-layer Cauchy-dispersion. Pictures were made by the CompleteEASE v. 5.15 software.

**Figure 8:**
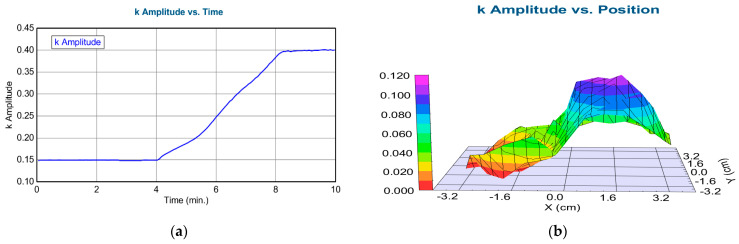
(**a**) The imaginary part of the refractive index (k Amplitude) as a function of time for highly conductive Si in the liquid cell during coloration (time-scan, simple 2-layer Cauchy model). From 0–4 min, there is low absorption, however, from 4–8 min, there is a growing absorption; (**b**) Map of the k parameter after coloration (simple 1-layer Cauchy-model). Pictures were made by the CompleteEASE v. 5.15 software.

After the validation of the SE method (we can determine the composition of the layer), we performed an in situ electrochromic measurement, shown in Figure 3. We could measure only at the central point of the highly conductive 3-inch Si-wafer. Figure 7 shows a typical example of one measured spectra pair with the model calculation based on the optical model shown on the right side. The low MSE value shows that the optical model is good. We could follow the process by calculating the change of the k parameter, as shown in Figure 8a and Table 2.

After the coloration process, we could map the colorized layer using a simple one-layer Cauchy dispersion optical model. Note that this is not the same model as it was used in the in situ measurement. We used the k Amplitude parameter of the Cauchy model as an indicator of the electrochromic effectiveness (the change in the light absorption for the same electric charge), i.e., the higher the k, the more effective the light absorption at that composition for the same electric charge.

We see a maximum value (maximum light absorption) around 1 cm, as shown in Table 3. Comparing these results with Figure 6 shows that the optimal composition is at (30%)TiO_2_–(70%)SnO_2_.

## 5. Conclusions

We could optimize the electrochromic properties of mixed titanium oxide and tin oxide layer deposited by reactive sputtering. We prepared combinatorial samples by magnetron sputtering. These samples were mapped (composition and thickness maps) via spectroscopic ellipsometry, which is a rapid, cost-effective, and contactless (non-destructive) method. The selection between the suitable optical models [the Bruggeman Effective Medium Approximation (BEMA) vs. the 2-Tauc–Lorentz multiple oscillator model (2T–L)] was conducted according to the process parameters. We have shown that in the case of molecular-level mixed layers, 2T–L is better than the BEMA optical models. We have shown that the optimal composition is at (30%)TiO_2_–(70%)SnO_2_.

## Figures and Tables

**Table 1:** Mean Squared Error (MSE) and Thickness values from EMA and 2T–L modelling.

X (cm)	MSE from EMA	MSE from 2-TL	Thickness [nm] from EMA	Thickness [nm] from 2-TL
0	29.8	31.0	549.2	545.3
1	35.2	35.6	516.7	515.7
2	37.8	37.0	485.2	482.5
3	40.3	40.5	457.1	455.7
4	40.7	39.8	433.3	433.2
5	43.7	44.1	412.5	413.4
6	47.4	46.8	395.6	397.1
7	51.8	510	380.4	384.2
8	52.2	49.9	367.8	372.7
9	52.9	48.8	359.7	364.8
10	53.3	48.5	352.8	359.1
11	57.1	49.7	348.1	357.6
12	59.2	50.2	346.8	357.9
13	58.5	48.8	349.8	360.9
14	58.7	46.5	356.5	368.3
15	59.9	44.4	366.5	381.4
16	58.1	41.3	379.8	395.2
17	54.6	38.0	395.4	410.1
18	50.3	34.9	412.3	426.2
19	46.1	32.5	431.1	443.7
20	42.0	28.9	446.1	457.9

**Table 2:** Cauchy parameter k Amplitude vs. Time at the center point during the colorization process.

Time (s)	k Amplitude	k Amplitude Error
0	0.149	0.001
60	0.149	0.001
120	0.149	0.001
180	0.148	0.001
240	0.149	0.001
270	0.169	0.001
300	0.188	0.002
330	0.211	0.002
360	0.246	0.0025
390	0.288	0.003
420	0.317	0.003
450	0.346	0.0035
480	0.386	0.004
510	0.397	0.004
540	0.399	0.004
570	0.400	0.004
600	0.400	0.0045

**Table 3:** k Amplitude vs. Position at the center line after the colorization in the dry state.

X (cm)	k Amplitude (Error ± 0.005)
−3.5	0.0002
−3	0.0025
−2.5	0.044
−2	0.004
−1.5	0.015
−1	0.025
−0.5	0.056
0	0.041
0.5	0.092
1	0.105
1.5	0.075
2	0.054
2.5	0.079
3	0.041
3. 5	0.039
